# Supplementary material for: The Inflammatory Cytokine IL-3 Hampers Cardioprotection Mediated by Endothelial Cell-Derived Extracellular Vesicles Possibly via Their Protein Cargo
Source: Cells. 2020 Dec 23;10(1):13. doi: 10.3390/cells10010013 (PMC7822476; doi:10.3390/cells10010013)
Supplement: Supplementary file 1 [file cells-10-00013-s001.pdf]

SUPPLEMENTARY MATERIALS

The inflammatory cytokine IL-3 hampers cardio-protection mediated by endothelial cell-derived extracellular vesicles possibly via their protein cargo

Claudia Penna[1], Saveria Femminò[2], Marta Tapparo[2], Tatiana Lopatina[2], Kari Espolin Fladmark[3], Stefano Comità[1], Francesco Ravera[2], Giuseppe Alloatti[4], Ilaria Giusti[5], Vincenza Dolo[5], Giovanni Camussi[2], Pasquale Pagliaro[1] Maria Felice Brizzi[2]

[1]Department of Clinical and Biological Sciences, University of Turin. Regione Gonzole 10, 10043, Orbassano (TO), Italy

[2]Department of Medical Sciences, University of Turin. Corso Dogliotti 14, 10126, Turin, Italy

[3]Department of Biological Science, University of Bergen, Thormohlensgt. 55, 5020 Bergen, Norway

[4]Uni-Astiss, Polo Universitario Rita Levi Montalcini, Asti, Italy

[5] Department of Life, Health and Environmental Sciences, University of L'Aquila, Via Vetoio - Coppito 2, 67100 L'Aquila, Italy

Figure S1

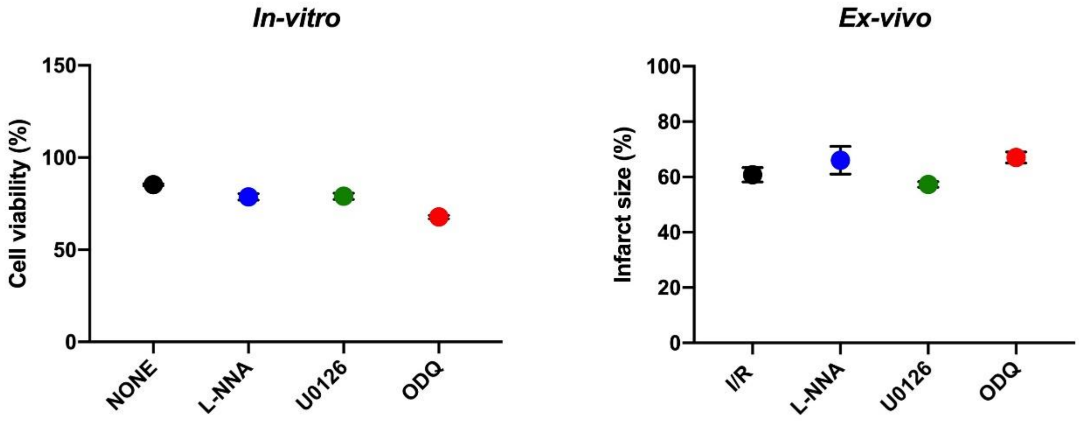

Figure S1. Left panel: HMEC-1 treated with the indicated inhibitors were subjected to H/R condition and analyzed for cell viability by MTT Assay performed on H9c2 (NONE corresponds to the H/R). **Right panel:** Infarct size in isolated rat hearts exposed to the indicated inhibitors (n=3/each group).

Table S1.

Majority protein IDs	Protein names	Gene names	LFQ intensity eEV (log2)	LFQ intensity eEV-IL-3 (log2)	Student's t-test q-value	Delta
P01023	Alpha-2-macroglobulin	A2M	23.581	21.417	0.000701	-2.164
P49588;Q8BGQ7	Alanine--tRNA ligase, cytoplasmic	AARS	21.750	25.617	0.000528	3.867
P33527	Multidrug resistance-associated protein 1	ABCC1	21.353	26.138	0.000000	4.785
Q9P1F3	Costars family protein ABRACL	ABRACL	20.112	25.480	0.000000	5.368
P49748	Very long-chain specific acyl-CoA dehydrogenase, mitochondrial	ACADVL	24.630	28.261	0.000581	3.631
P24752	Acetyl-CoA acetyltransferase, mitochondrial	ACAT1	19.779	27.808	0.000000	8.029
Q99798	Aconitate hydratase, mitochondrial	ACO2	19.697	27.635	0.000000	7.938
O00154;Q91V12	Cytosolic acyl coenzyme A thioester hydrolase	ACOT7	22.694	25.308	0.000353	2.614
P68133;P68032;P63267;P62736	Actin, alpha skeletal muscle;Actin, alpha cardiac muscle 1;Actin, gamma-enteric smooth muscle;Actin, aortic smooth muscle	ACTA1	29.247	31.108	0.001997	1.861
P63261	Actin, cytoplasmic 2;Actin, cytoplasmic 2, N-terminally processed	ACTG1	33.162	34.792	0.003469	1.630
P12814	Alpha-actinin-1	ACTN1	29.257	31.467	0.000614	2.210
O43707	Alpha-actinin-4	ACTN4	27.889	30.336	0.000399	2.447
Q76LX8	A disintegrin and metalloproteinase with thrombospondin motifs 13	ADAMTS13	24.434	0.000	0.000391	-24.434
P30566	Adenylosuccinate lyase	ADSL	21.723	24.490	0.000411	2.767
Q09666	Neuroblast differentiation-associated protein AHNAK	AHNAK	23.746	26.315	0.001923	2.569
P00568	Adenylate kinase isoenzyme 1	AK1	23.056	26.754	0.000623	3.698
P15121	Aldose reductase	AKR1B1	25.205	26.815	0.004127	1.611
O43488;Q8NHP1;Q8CG76;Q8CG45	Aflatoxin B1 aldehyde reductase member 2;Aflatoxin B1 aldehyde reductase member 4	AKR7A2	21.642	23.218	0.004062	1.576
P54886	Delta-1-pyrroline-5-carboxylate synthase;Glutamate 5-kinase;Gamma-glutamyl phosphate reductase	ALDH18A1	29.322	27.642	0.004459	-1.680
P00352	Retinal dehydrogenase 1	ALDH1A1	25.798	27.606	0.003047	1.808
P05091	Aldehyde dehydrogenase, mitochondrial	ALDH2	23.953	27.652	0.000638	3.700

P49419	Alpha-aminoadipic semialdehyde dehydrogenase	ALDH7A1	24.596	27.534	0.000380	2.938
P04075;P00883;A5A6I5	Fructose-bisphosphate aldolase A	ALDOA	29.031	30.856	0.001769	1.825
P09972	Fructose-bisphosphate aldolase C	ALDOC	25.633	27.134	0.004688	1.501
Q86V81	THO complex subunit 4	ALYREF	0.000	24.473	0.000410	24.473
O15123	Angiopoietin-2	ANGPT2	24.108	26.091	0.001923	1.983
P39687	Acidic leucine-rich nuclear phosphoprotein 32 family member A	ANP32A	21.387	26.841	0.000248	5.454
Q92688	Acidic leucine-rich nuclear phosphoprotein 32 family member B	ANP32B	22.699	24.325	0.004121	1.626
P15144	Aminopeptidase N	ANPEP	28.667	30.352	0.003376	1.685
Q10567;P52303	AP-1 complex subunit beta-1	AP1B1	21.292	23.448	0.000847	2.156
P27695;A2T6Y4;A1YFZ3;A2T7I6;A1YES6	DNA-(apurinic or apyrimidinic site) lyase;DNA-(apurinic or apyrimidinic site) lyase, mitochondrial	APEX1	0.000	26.890	0.000431	26.890
Q06481;P15943	Amyloid-like protein 2	APLP2	0.000	24.329	0.000373	24.329
Q9HDC9	Adipocyte plasma membrane-associated protein	APMAP	24.191	29.251	0.000000	5.060
P04114	Apolipoprotein B-100;Apolipoprotein B-48	APOB	26.681	23.025	0.000657	-3.656
P19034;P18658;P02655	Apolipoprotein C-II	APOC2	25.486	22.931	0.000652	-2.555
P53601;P05067;P12023	Amyloid beta A4 protein	APP	0.000	26.235	0.000632	26.235
P48444	Coatomer subunit delta	ARCN1	22.191	25.855	0.000603	3.664
P18085	ADP-ribosylation factor 4	ARF4	25.787	27.550	0.003467	1.762
P52565	Rho GDP-dissociation inhibitor 1	ARHGDIA	26.328	28.556	0.000639	2.228
P52566	Rho GDP-dissociation inhibitor 2	ARHGDIB	25.813	27.589	0.002238	1.776
Q15041	ADP-ribosylation factor-like protein 6-interacting protein 1	ARL6IP1	23.324	26.833	0.000530	3.509
O75915	PRA1 family protein 3	ARL6IP5	23.612	27.581	0.000763	3.969
Q9NVJ2;Q96BM9	ADP-ribosylation factor-like protein 8B;ADP-ribosylation factor-like protein 8A	ARL8A	22.948	25.390	0.000624	2.443
O15144	Actin-related protein 2/3 complex subunit 2	ARPC2	26.408	27.800	0.005110	1.392
O15511	Actin-related protein 2/3 complex subunit 5	ARPC5	23.981	26.093	0.000653	2.112
Q9BPX5	Actin-related protein 2/3 complex subunit 5-like protein	ARPC5L	22.106	23.657	0.004111	1.551

O43681;Q6IQE5	ATPase ASNA1;ATPase asna1	ASNA1	22.462	24.885	0.000599	2.423
P00966	Argininosuccinate synthase	ASS1	24.656	0.000	0.000575	-24.656
Q9NVI7	ATPase family AAA domain-containing protein 3A	ATAD3A	0.000	25.532	0.004122	25.532
Q6DD88	Atlastin-3	ATL3	22.839	28.423	0.000225	5.584
P05023	Sodium/potassium-transporting ATPase subunit alpha-1	ATP1A1	27.348	29.379	0.001812	2.031
P54709	Sodium/potassium-transporting ATPase subunit beta-3	ATP1B3	24.549	27.666	0.000378	3.118
P16615	Sarcoplasmic/endoplasmic reticulum calcium ATPase 2	ATP2A2	0.000	29.077	0.000000	29.077
P23634	Plasma membrane calcium-transporting ATPase 4	ATP2B4	22.906	27.945	0.000660	5.039
A5A6H5;P25705	ATP synthase subunit alpha, mitochondrial	ATP5A1	26.397	31.940	0.000000	5.544
P06576;P00829	ATP synthase subunit beta, mitochondrial	ATP5B	27.701	33.105	0.000000	5.404
P36542	ATP synthase subunit gamma, mitochondrial	ATP5C1	22.695	29.123	0.000224	6.428
P30049	ATP synthase subunit delta, mitochondrial	ATP5D	0.000	27.182	0.001977	27.182
P24539	ATP synthase F(0) complex subunit B1, mitochondrial	ATP5F1	18.173	28.838	0.000000	10.665
O75947	ATP synthase subunit d, mitochondrial	ATP5H	0.000	29.104	0.000000	29.104
P56134	ATP synthase subunit f, mitochondrial	ATP5J2	0.000	28.081	0.000000	28.081
P48047	ATP synthase subunit O, mitochondrial	ATP5O	20.832	29.337	0.000000	8.505
P38606	V-type proton ATPase catalytic subunit A	ATP6V1A	24.634	25.904	0.008483	1.269
P61769	Beta-2-microglobulin;Beta-2-microglobulin form pI 5.3	B2M	21.975	27.588	0.000000	5.613
O43505	N-acetyllactosaminide beta-1,3-N-acetylglucosaminyltransferase	B3GNT1	26.073	23.710	0.000593	-2.364
O75531	Barrier-to-autointegration factor;Barrier-to-autointegration factor, N-terminally processed	BANF1	23.619	26.352	0.000569	2.733
P80723	Brain acid soluble protein 1	BASP1	24.475	26.071	0.003882	1.596
P51572	B-cell receptor-associated protein 31	BCAP31	23.106	28.017	0.000000	4.911
P54687	Branched-chain-amino-acid aminotransferase, cytosolic	BCAT1	23.448	25.777	0.001800	2.329
P55957	BH3-interacting domain death agonist;BH3-interacting domain death agonist p15;BH3-interacting domain death agonist p13;BH3-interacting domain death agonist p11	BID	18.745	25.187	0.000000	6.441
P35613	Basigin	BSG	29.360	31.337	0.000974	1.978
P20290	Transcription factor BTF3	BTF3	24.346	25.991	0.009790	1.645

Q7L1Q6	Basic leucine zipper and W2 domain-containing protein 1	BZW1	23.593	25.078	0.005288	1.485
Q9Y224	UPF0568 protein C14orf166	C14orf 166	23.213	26.350	0.000375	3.137
Q969H8	UPF0556 protein C19orf10	C19orf 10	20.743	28.564	0.000000	7.821
Q07021	Complement component 1 Q subcomponent-binding protein, mitochondrial	C1QBP	21.575	28.513	0.000247	6.939
Q9BXJ4	Complement C1q tumor necrosis factor-related protein 3	C1QTN F3	24.696	21.062	0.000508	-3.634
Q8CG16	Complement C1r-A subcomponent	C1RA	25.217	22.037	0.000537	-3.181
P13671	Complement component C6	C6	26.368	23.767	0.000676	-2.601
P07358	Complement component C8 beta chain	C8B	26.590	24.065	0.000452	-2.524
Q9HB71	Calcyclin-binding protein	CACY BP	23.054	24.733	0.005156	1.679
Q6PI52;P62158	Calmodulin	CALM 1	27.465	30.255	0.000433	2.791
P27797	Calreticulin	CALR	27.509	32.782	0.000000	5.273
O43852	Calumenin	CALU	24.531	30.936	0.000000	6.405
P27824;P35565	Calnexin	CANX	25.049	30.894	0.000000	5.844
P07384	Calpain-1 catalytic subunit	CAPN1	24.172	26.440	0.000853	2.268
P17655	Calpain-2 catalytic subunit	CAPN2	26.430	28.015	0.004133	1.585
P04632	Calpain small subunit 1	CAPNS 1	26.309	27.795	0.004306	1.486
P52907	F-actin-capping protein subunit alpha-1	CAPZA 1	26.014	27.928	0.001803	1.914
P47755	F-actin-capping protein subunit alpha-2	CAPZA 2	23.647	26.119	0.000643	2.472
P47756	F-actin-capping protein subunit beta	CAPZB	24.361	27.717	0.000409	3.355
P04040	Catalase	CAT	23.157	26.447	0.000364	3.290
Q03135	Caveolin-1	CAV1	26.783	28.180	0.008192	1.398
Q13185	Chromobox protein homolog 3	CBX3	22.075	25.267	0.000439	3.192
Q6YHK3	CD109 antigen	CD109	25.661	27.064	0.004915	1.403
P48509	CD151 antigen	CD151	23.141	24.654	0.008522	1.513
Q5ZPR3	CD276 antigen	CD276	23.852	26.425	0.000626	2.572
P13987	CD59 glycoprotein	CD59	27.613	29.324	0.003887	1.711
Q9NPY3	Complement component C1q receptor	CD93	21.531	23.938	0.001405	2.407
P14209	CD99 antigen	CD99	22.579	24.809	0.002108	2.230
P55290;Q9WTR 5	Cadherin-13	CDH13	24.990	27.140	0.000650	2.150
P33151	Cadherin-5	CDH5	22.784	24.886	0.001426	2.103
P23528	Cofilin-1	CFL1	28.902	31.750	0.000382	2.848

Q99653	Calcineurin B homologous protein 1	CHP1	21.295	23.264	0.002101	1.969
Q07065	Cytoskeleton-associated protein 4	CKAP4	22.637	31.836	0.000000	9.199
O00299	Chloride intracellular channel protein 1	CLIC1	27.253	28.835	0.003994	1.581
Q9Y696	Chloride intracellular channel protein 4	CLIC4	25.481	27.979	0.000577	2.497
P10909	Clusterin;Clusterin beta chain;Clusterin alpha chain	CLU	27.485	25.582	0.001400	-1.903
P30085	UMP-CMP kinase	CMPK 1	20.257	25.531	0.000000	5.274
Q96KP4	Cytosolic non-specific dipeptidase	CNDP2	21.994	26.542	0.000238	4.548
Q99439	Calponin-2	CNN2	23.601	25.523	0.003146	1.922
Q12860	Contactin-1	CNTN1	25.123	20.055	0.000706	-5.068
Q99715	Collagen alpha-1(XII) chain	COL12 A1	28.643	26.620	0.003086	-2.023
P02465;P08123	Collagen alpha-2(I) chain	COL1A 2	25.723	24.269	0.006790	-1.454
P02462	Collagen alpha-1(IV) chain;Arresten	COL4A 1	24.321	26.840	0.002909	2.518
P12109	Collagen alpha-1(VI) chain	COL6A 1	28.069	25.289	0.000502	-2.781
P12111	Collagen alpha-3(VI) chain	COL6A 3	25.240	22.756	0.000637	-2.485
Q8NBJ5	Procollagen galactosyltransferase 1	COLG ALT1	22.390	28.870	0.000234	6.479
P53618	Coatomer subunit beta	COPB1	25.954	27.740	0.004045	1.786
O14579	Coatomer subunit epsilon	COPE	23.589	26.854	0.000402	3.265
P61923	Coatomer subunit zeta-1	COPZ1	23.762	25.604	0.003171	1.842
Q9BR76	Coronin-1B	CORO1 B	22.292	25.433	0.000395	3.141
Q9ULV4	Coronin-1C	CORO1 C	24.434	26.717	0.000849	2.283
P13073	Cytochrome c oxidase subunit 4 isoform 1, mitochondrial	COX4I 1	0.000	28.065	0.000000	28.065
P20674	Cytochrome c oxidase subunit 5A, mitochondrial	COX5 A	0.000	27.688	0.000000	27.688
P10606	Cytochrome c oxidase subunit 5B, mitochondrial	COX5B	21.459	27.536	0.000714	6.077
P00450	Ceruloplasmin	CP	25.487	22.481	0.000457	-3.005
Q99829	Copine-1	CPNE1	22.251	24.055	0.004219	1.804
P52943	Cysteine-rich protein 2	CRIP2	0.000	25.396	0.000366	25.396
O75390	Citrate synthase, mitochondrial	CS	26.660	29.424	0.000403	2.764
P55060	Exportin-2	CSE1L	23.928	27.620	0.000440	3.692
P21291	Cysteine and glycine-rich protein 1	CSRP1	24.331	27.266	0.000444	2.935
P04080	Cystatin-B	CSTB	0.000	26.385	0.000684	26.385
P29279	Connective tissue growth factor	CTGF	29.689	28.303	0.005174	-1.387
P35221	Catenin alpha-1	CTNN A1	26.166	28.187	0.002051	2.021

P35222	Catenin beta-1	CTNNB1	23.929	26.631	0.000592	2.702
P10619	Lysosomal protective protein;Lysosomal protective protein 32 kDa chain;Lysosomal protective protein 20 kDa chain	CTSA	20.467	26.154	0.000000	5.687
P07858	Cathepsin B;Cathepsin B light chain;Cathepsin B heavy chain	CTSB	26.972	30.121	0.000557	3.149
P53634	Dipeptidyl peptidase 1;Dipeptidyl peptidase 1 exclusion domain chain;Dipeptidyl peptidase 1 heavy chain;Dipeptidyl peptidase 1 light chain	CTSC	25.356	28.444	0.000458	3.089
P07339	Cathepsin D;Cathepsin D light chain;Cathepsin D heavy chain	CTSD	24.748	29.588	0.000000	4.841
Q9UBR2	Cathepsin Z	CTSZ	19.638	27.172	0.000000	7.534
O43169	Cytochrome b5 type B	CYB5B	22.660	27.979	0.000000	5.319
P00387	NADH-cytochrome b5 reductase 3;NADH-cytochrome b5 reductase 3 membrane-bound form;NADH-cytochrome b5 reductase 3 soluble form	CYB5R3	20.873	28.603	0.000000	7.730
P99999	Cytochrome c	CYCS	22.128	27.492	0.000000	5.363
O00622;A5A6L1;P18406	Protein CYR61	CYR61	28.648	26.963	0.003654	-1.685
Q13561	Dynactin subunit 2	DCTN2	22.145	25.059	0.000574	2.915
O95865	N(G),N(G)-dimethylarginine dimethylaminohydrolase 2	DDAH2	23.054	25.225	0.001542	2.171
Q16531	DNA damage-binding protein 1	DDB1	25.122	26.744	0.004050	1.622
P39656;O54734	Dolichyl-diphosphooligosaccharide--protein glycosyltransferase 48 kDa subunit	DDOST	20.055	29.662	0.000000	9.608
Q92499	ATP-dependent RNA helicase DDX1	DDX1	24.041	25.947	0.001806	1.906
P60024;Q9Z1N5;Q13838;O00148	Spliceosome RNA helicase Ddx39b;Spliceosome RNA helicase DDX39B;ATP-dependent RNA helicase DDX39A	DDX39B	24.473	27.361	0.000505	2.888
O00571;Q62095;Q62167;O15523	ATP-dependent RNA helicase DDX3X;ATP-dependent RNA helicase DDX3Y	DDX3X	24.091	25.716	0.007265	1.626
Q16698	2,4-dienoyl-CoA reductase, mitochondrial	DECR1	21.912	26.223	0.000772	4.312
Q6IQ26	DENN domain-containing protein 5A	DENND5A	25.652	24.187	0.005621	-1.465
Q9Y315	Putative deoxyribose-phosphate aldolase	DERA	25.412	23.957	0.004676	-1.456
P09622;P09623	Dihydrolipoyl dehydrogenase, mitochondrial	DLD	20.292	28.423	0.000000	8.131

P36957	Dihydrolipoyllysine-residue succinyltransferase component of 2-oxoglutarate dehydrogenase complex, mitochondrial	DLST	19.369	27.862	0.000000	8.493
Q8IXB1	DnaJ homolog subfamily C member 10	DNAJC 10	19.783	27.636	0.000000	7.853
O75165	DnaJ homolog subfamily C member 13	DNAJC 13	24.827	23.386	0.005261	-1.442
Q13217;Q27968 ;Q91YW3	DnaJ homolog subfamily C member 3	DNAJC 3	19.819	27.178	0.000000	7.360
Q9H3Z4	DnaJ homolog subfamily C member 5	DNAJC 5	22.102	25.199	0.000424	3.097
P49184	Deoxyribonuclease-1-like 1	DNAS E1L1	0.000	22.445	0.006038	22.445
Q9NY33	Dipeptidyl peptidase 3	DPP3	20.855	26.037	0.000000	5.182
Q16555	Dihydropyrimidinase-related protein 2	DPYSL 2	27.835	29.170	0.006010	1.335
Q14195	Dihydropyrimidinase-related protein 3	DPYSL 3	24.291	26.658	0.000514	2.367
P60981	Destrin	DSTN	24.253	26.584	0.000976	2.331
Q13409;O88487	Cytoplasmic dynein 1 intermediate chain 2	DYNC 1I2	22.928	25.907	0.000342	2.978
Q8NCM8;Q45V K7	Cytoplasmic dynein 2 heavy chain 1	DYNC 2H1	25.038	22.677	0.000604	-2.362
Q9NP97;Q8TF0 9	Dynein light chain roadblock-type 1;Dynein light chain roadblock-type 2	DYNL RB1	0.000	25.261	0.000485	25.261
P63172	Dynein light chain Tctex-type 1	DYNL T1	0.000	24.419	0.004056	24.419
O75923	Dysferlin	DYSF	24.521	29.682	0.000781	5.161
P42892;P42891	Endothelin-converting enzyme 1	ECE1	24.897	27.782	0.000347	2.884
Q13011	Delta(3,5)-Delta(2,4)-dienoyl-CoA isomerase, mitochondrial	ECH1	20.221	26.771	0.000000	6.550
P30084	Enoyl-CoA hydratase, mitochondrial	ECHS1	19.139	28.268	0.000000	9.128
Q61508	Extracellular matrix protein 1	ECM1	24.525	0.000	0.004126	-24.525
P68104;Q5VTE 0;P62630;P1012 6	Elongation factor 1-alpha 1;Putative elongation factor 1-alpha-like 3	EEF1A 1	30.513	31.888	0.005176	1.376
P24534	Elongation factor 1-beta	EEF1B 2	23.245	27.600	0.000673	4.355
P29692	Elongation factor 1-delta	EEF1D	24.139	27.574	0.000359	3.436
P13639;P58252	Elongation factor 2	EEF2	28.890	30.466	0.003648	1.576
Q9NZN3	EH domain-containing protein 3	EHD3	24.006	0.000	0.003156	-24.006

P05198	Eukaryotic translation initiation factor 2 subunit 1	EIF2S1	24.522	26.476	0.001907	1.955
P20042	Eukaryotic translation initiation factor 2 subunit 2	EIF2S2	22.831	25.111	0.001421	2.281
P41091;Q9Z0N1;Q2VIR3;Q9Z0N2	Eukaryotic translation initiation factor 2 subunit 3;Eukaryotic translation initiation factor 2 subunit 3, X-linked;Putative eukaryotic translation initiation factor 2 subunit 3-like protein;Eukaryotic translation initiation factor 2 subunit 3, Y-linked	EIF2S3	25.571	27.267	0.003151	1.696
P55884	Eukaryotic translation initiation factor 3 subunit B	EIF3B	23.542	25.268	0.003989	1.726
Q13347	Eukaryotic translation initiation factor 3 subunit I	EIF3I	23.741	25.425	0.003473	1.685
Q9UBQ5	Eukaryotic translation initiation factor 3 subunit K	EIF3K	22.891	24.634	0.005275	1.743
P60842;A5A6N4	Eukaryotic initiation factor 4A-I	EIF4A1	26.694	28.350	0.003455	1.656
Q04637;P41110	Eukaryotic translation initiation factor 4 gamma 1	EIF4G1	0.000	24.838	0.005985	24.838
P63241;Q6IS14;Q9GZV4;Q8BGY2	Eukaryotic translation initiation factor 5A-1;Eukaryotic translation initiation factor 5A-1-like;Eukaryotic translation initiation factor 5A-2	EIF5A	26.382	28.587	0.000576	2.205
P56537;O55135	Eukaryotic translation initiation factor 6	EIF6	23.701	26.338	0.000396	2.637
Q15717	ELAV-like protein 1	ELAVL1	21.055	24.659	0.000574	3.605
P17813	Endoglin	ENG	26.808	28.803	0.001608	1.995
Q9XSJ4;P17182	Alpha-enolase	ENO1	27.855	24.829	0.000386	-3.026
P06733	Alpha-enolase	ENO1	29.995	31.990	0.001372	1.994
Q5NDL2	EGF domain-specific O-linked N-acetylglucosamine transferase	EOGT	23.494	25.988	0.001606	2.494
P29317	Ephrin type-A receptor 2	EPHA2	23.706	25.439	0.005204	1.733
Q9NZ08	Endoplasmic reticulum aminopeptidase 1	ERAP1	22.474	28.172	0.000242	5.698
Q98874;P84090	Enhancer of rudimentary homolog	ERH	22.189	25.987	0.000754	3.798
Q96HE7	ERO1-like protein alpha	ERO1L	0.000	28.257	0.000000	28.257
P30040;P81623	Endoplasmic reticulum resident protein 29	ERP29	17.758	28.793	0.000000	11.035
Q9BS26	Endoplasmic reticulum resident protein 44	ERP44	19.973	29.038	0.000000	9.065
P10768	S-formylglutathione hydrolase	ESD	24.313	26.225	0.001794	1.912

Q9NQ30	Endothelial cell-specific molecule 1	ESM1	0.000	23.051	0.006045	23.051
Q9BSJ8	Extended synaptotagmin-1	ESYT1	19.497	27.969	0.000000	8.472
P62495	Eukaryotic peptide chain release factor subunit 1	ETF1	21.538	24.457	0.000383	2.920
P13804	Electron transfer flavoprotein subunit alpha, mitochondrial	ETFA	21.422	26.641	0.000000	5.219
Q96A65	Exocyst complex component 4	EXOC4	26.603	24.858	0.004105	-1.745
P00742	Coagulation factor X;Factor X light chain;Factor X heavy chain;Activated factor Xa heavy chain	F10	28.121	25.140	0.000436	-2.981
Q9Y624	Junctional adhesion molecule A	F11R	23.111	25.651	0.000603	2.540
P00488	Coagulation factor XIII A chain	F13A1	27.088	23.094	0.000710	-3.993
P15090;A6YLM6	Fatty acid-binding protein, adipocyte	FABP4	21.575	26.181	0.000252	4.606
Q01469	Fatty acid-binding protein, epidermal	FABP5	26.814	29.785	0.000468	2.971
Q96TA1	Niban-like protein 1	FAM129B	0.000	26.147	0.000650	26.147
Q9BRX8	Redox-regulatory protein FAM213A	FAM213A	21.163	29.218	0.000000	8.055
Q9NSD9	Phenylalanine--tRNA ligase beta subunit	FARSB	23.917	25.448	0.004874	1.530
Q6V0I7	Protocadherin Fat 4	FAT4	23.252	0.000	0.008511	-23.252
P14324	Farnesyl pyrophosphate synthase	FDPS	22.068	26.256	0.000240	4.188
P22570	NADPH:adrenodoxin oxidoreductase, mitochondrial	FDXR	21.613	26.074	0.000670	4.461
P02671	Fibrinogen alpha chain;Fibrinopeptide A;Fibrinogen alpha chain	FGA	25.650	21.556	0.000663	-4.094
P07954	Fumarate hydratase, mitochondrial	FH	24.330	28.611	0.000776	4.280
Q14192	Four and a half LIM domains protein 2	FHL2	23.692	26.205	0.000667	2.513
Q96AY3	Peptidyl-prolyl cis-trans isomerase FKBP10	FKBP10	0.000	28.524	0.000000	28.524
P62942	Peptidyl-prolyl cis-trans isomerase FKBP1A	FKBP1A	24.559	29.237	0.000232	4.678
Q02790	Peptidyl-prolyl cis-trans isomerase FKBP4;Peptidyl-prolyl cis-trans isomerase FKBP4, N-terminally processed	FKBP4	23.178	25.438	0.000597	2.260
O95302	Peptidyl-prolyl cis-trans isomerase FKBP9	FKBP9	21.561	26.946	0.000750	5.385
Q13045	Protein flightless-1 homolog	FLII	20.958	25.351	0.000746	4.392
O75369	Filamin-B	FLNB	29.357	32.089	0.000415	2.732
Q14315	Filamin-C	FLNC	21.530	28.149	0.000000	6.619
Q7YR41;O75955	Flotillin-1	FLOT1	0.000	24.355	0.005163	24.355

Q06828	Fibromodulin	FMOD	24.630	0.000	0.000351	-24.630
P02751	Fibronectin;Anastellin;Ugl-Y1;Ugl-Y2;Ugl-Y3	FN1	34.143	31.261	0.000361	-2.882
Q16658;Q61553	Fascin	FSCN1	28.195	29.985	0.002245	1.790
Q13283	Ras GTPase-activating protein-binding protein 1	G3BP1	22.744	26.193	0.000414	3.449
P11413	Glucose-6-phosphate 1-dehydrogenase	G6PD	23.701	26.439	0.000390	2.737
P10253	Lysosomal alpha-glucosidase;76 kDa lysosomal alpha-glucosidase;70 kDa lysosomal alpha-glucosidase	GAA	23.128	27.129	0.000506	4.000
Q10472	Polypeptide N-acetylgalactosaminyltransferase 1;Polypeptide N-acetylgalactosaminyltransferase 1 soluble form	GALNT1	20.221	27.218	0.000000	6.997
Q14697	Neutral alpha-glucosidase AB	GANAB	25.603	31.724	0.000000	6.121
P04797	Glyceraldehyde-3-phosphate dehydrogenase	GAPDH	26.808	23.461	0.000422	-3.347
P41250	Glycine--tRNA ligase	GARS	24.623	27.534	0.000367	2.911
P60028;P31150	Rab GDP dissociation inhibitor alpha	GDI1	22.276	25.004	0.000372	2.728
P50395	Rab GDP dissociation inhibitor beta	GDI2	27.502	29.235	0.003061	1.734
Q92820	Gamma-glutamyl hydrolase	GGH	24.418	27.112	0.000356	2.695
Q9NUV9	GTPase IMAP family member 4	GIMAP4	23.412	25.879	0.000504	2.466
P16278	Beta-galactosidase	GLB1	21.741	25.596	0.000460	3.856
Q92896	Golgi apparatus protein 1	GLG1	20.597	28.975	0.000000	8.378
Q9HC38	Glyoxalase domain-containing protein 4	GLOD4	22.897	24.541	0.004453	1.644
O76003	Glutaredoxin-3	GLRX3	22.402	26.067	0.000653	3.665
P00367;P00366;P49448	Glutamate dehydrogenase 1, mitochondrial;Glutamate dehydrogenase 2, mitochondrial	GLUD1	20.476	29.902	0.000000	9.427
P29992;O95837	Guanine nucleotide-binding protein subunit alpha-11;Guanine nucleotide-binding protein subunit alpha-14	GNA11	23.098	24.430	0.009966	1.332
P04899	Guanine nucleotide-binding protein G(i) subunit alpha-2	GNAI2	26.413	28.610	0.001395	2.197
P50148	Guanine nucleotide-binding protein G(q) subunit alpha	GNAQ	23.228	24.961	0.004110	1.733
P62873	Guanine nucleotide-binding protein G(I)/G(S)/G(T) subunit beta-1	GNB1	26.490	27.915	0.005116	1.424
Q9UBI6	Guanine nucleotide-binding protein G(I)/G(S)/G(O) subunit gamma-12	GNG12	23.707	26.414	0.000579	2.707
Q08378	Golgin subfamily A member 3	GOLGA3	25.178	22.962	0.001468	-2.216

Q9H8Y8	Golgi reassembly-stacking protein 2	GORASP2	21.957	24.211	0.000621	2.254
P06744	Glucose-6-phosphate isomerase	GPI	26.334	28.349	0.000747	2.015
Q8IZF2	Probable G-protein coupled receptor 116	GPR116	20.967	25.174	0.000562	4.208
P07203	Glutathione peroxidase 1	GPX1	25.006	26.798	0.002058	1.792
Q9UBQ7	Glyoxylate reductase/hydroxypyruvate reductase	GRHPR	21.657	25.279	0.000478	3.622
P06396	Gelsolin	GSN	20.751	24.839	0.000680	4.087
P15170;Q8IYD1;Q149F3	Eukaryotic peptide chain release factor GTP-binding subunit ERF3A;Eukaryotic peptide chain release factor GTP-binding subunit ERF3B	GSPT1	22.924	24.658	0.004694	1.734
P00390	Glutathione reductase, mitochondrial	GSR	20.924	23.253	0.000662	2.328
P78417	Glutathione S-transferase omega-1	GSTO1	24.146	27.503	0.000532	3.357
P09211	Glutathione S-transferase P	GSTP1	27.285	30.190	0.000449	2.905
Q16836	Hydroxyacyl-coenzyme A dehydrogenase, mitochondrial	HADH	20.224	25.960	0.000000	5.735
P40939	Trifunctional enzyme subunit alpha, mitochondrial;Long-chain enoyl-CoA hydratase;Long chain 3-hydroxyacyl-CoA dehydrogenase	HADHA	21.499	30.947	0.000000	9.449
P55084	Trifunctional enzyme subunit beta, mitochondrial;3-ketoacyl-CoA thiolase	HADHB	24.178	29.923	0.000000	5.746
P12081	Histidine--tRNA ligase, cytoplasmic	HARS	21.140	25.913	0.000000	4.773
P51858	Hepatoma-derived growth factor	HDGF	22.694	25.625	0.000506	2.931
P06865	Beta-hexosaminidase subunit alpha	HEXA	23.069	26.996	0.000667	3.928
P07686	Beta-hexosaminidase subunit beta;Beta-hexosaminidase subunit beta chain B;Beta-hexosaminidase subunit beta chain A	HEXB	22.007	29.246	0.000000	7.238
Q96QV1	Hedgehog-interacting protein	HHIP	30.506	28.945	0.005268	-1.561
P49773	Histidine triad nucleotide-binding protein 1	HINT1	23.653	26.922	0.000416	3.269
P16401;P43276	Histone H1.5	HIST1H1B	24.989	27.342	0.000622	2.354
P10412;P15864;P43277;P16402	Histone H1.4;Histone H1.2;Histone H1.3	HIST1H1E	25.153	28.092	0.000443	2.939
P19367	Hexokinase-1	HK1	21.352	25.906	0.000786	4.554
P05534;P30447	HLA class I histocompatibility antigen, A-24 alpha chain;HLA class I histocompatibility antigen, A-23 alpha chain	HLA-A	22.294	23.869	0.005190	1.575

P04439;P30455;P30443;P13746;P16188;P30459;P10314	HLA class I histocompatibility antigen,	HLA-A	25.958	27.817	0.002474	1.860
Q29940;P30685;P30498;P30495;P30493;P30492;P30491;P30490;P30484;P30464;P18465;P18464;P10319;Q95365;Q29836;Q29718;P30480;P30475;P30462;P30460;P18463;P03989;P01889;Q31610;P30486;Q04826;P30488;P30487;P30485;P30483;P30481;P30479;P30461;P06140;P30382;P30510;P30504	HLA class I histocompatibility antigen,	HLA-B	22.839	25.724	0.000344	2.885
Q8TCT9	Minor histocompatibility antigen H13	HM13	0.000	27.403	0.000000	27.403
P17096	High mobility group protein HMG-I/HMG-Y	HMGA1	25.544	27.444	0.001611	1.900
P35914	Hydroxymethylglutaryl-CoA lyase, mitochondrial	HMGCL	21.872	24.208	0.001921	2.336
P30519	Heme oxygenase 2	HMOX2	20.369	27.373	0.000000	7.004
A5A6H4;P09651;Q32P51	Heterogeneous nuclear ribonucleoprotein A1;Heterogeneous nuclear ribonucleoprotein A1, N-terminally processed;Heterogeneous nuclear ribonucleoprotein A1-like 2	HNRNPA1	26.058	28.663	0.000360	2.604
P22626	Heterogeneous nuclear ribonucleoproteins A2/B1	HNRNPA2B1	26.347	28.746	0.000610	2.399
P51991	Heterogeneous nuclear ribonucleoprotein A3	HNRNPA3	22.503	25.758	0.000454	3.255
Q99729	Heterogeneous nuclear ribonucleoprotein A/B	HNRNPAB	22.573	25.366	0.000507	2.793
P07910	Heterogeneous nuclear ribonucleoproteins C1/C2	HNRNPC	25.817	27.775	0.003462	1.958
Q14103	Heterogeneous nuclear ribonucleoprotein D0	HNRNPD	26.809	28.756	0.001377	1.947
P52597	Heterogeneous nuclear ribonucleoprotein F;Heterogeneous nuclear ribonucleoprotein F, N-terminally processed	HNRNPF	22.759	26.059	0.000418	3.300
P31943;P70333;P55795	Heterogeneous nuclear ribonucleoprotein H;Heterogeneous nuclear	HNRNPH1	23.589	25.672	0.001758	2.083

	ribonucleoprotein H, N-terminally processed;Heterogeneous nuclear ribonucleoprotein H2;Heterogeneous nuclear ribonucleoprotein H2, N-terminally processed					
P61979;P61978	Heterogeneous nuclear ribonucleoprotein K	HNRNPK	26.277	28.275	0.001608	1.998
P14866	Heterogeneous nuclear ribonucleoprotein L	HNRNPL	24.636	27.322	0.000365	2.686
Q1KMD3	Heterogeneous nuclear ribonucleoprotein U-like protein 2	HNRNPUL2	23.294	25.192	0.002785	1.898
P00738;P00739	Haptoglobin;Haptoglobin alpha chain;Haptoglobin beta chain;Haptoglobin-related protein	HP	29.131	26.171	0.000355	-2.959
P37235;P84075;P84074	Hippocalcin-like protein 1;Neuron-specific calcium-binding protein hippocalcin	HPCAL1	20.963	23.991	0.000423	3.028
P32754	4-hydroxyphenylpyruvate dioxygenase	HPD	24.361	0.000	0.000653	-24.361
A5A6I1;P00492	Hypoxanthine-guanine phosphoribosyltransferase	HPRT1	20.044	23.122	0.000493	3.078
P08238;P11499	Heat shock protein HSP 90-beta	HSP90AB1	29.548	31.226	0.002794	1.678
P14625	Endoplasmin	HSP90B1	28.660	33.612	0.000000	4.951
P34932	Heat shock 70 kDa protein 4	HSPA4	24.606	27.693	0.000523	3.086
P11021;P20029	78 kDa glucose-regulated protein	HSPA5	28.109	33.414	0.000000	5.305
P11142;P63017	Heat shock cognate 71 kDa protein	HSPA8	29.705	31.093	0.005089	1.389
P38646	Stress-70 protein, mitochondrial	HSPA9	21.543	29.562	0.000000	8.019
P10809	60 kDa heat shock protein, mitochondrial	HSPD1	27.642	32.373	0.000000	4.731
P61604	10 kDa heat shock protein, mitochondrial	HSPE1	25.072	28.649	0.000576	3.577
P98160	Basement membrane-specific heparan sulfate proteoglycan core protein;Endorepellin;LG3 peptide	HSPG2	33.801	31.442	0.000661	-2.359
Q92598	Heat shock protein 105 kDa	HSPH1	21.036	23.324	0.001127	2.288
Q92743;F1N152	Serine protease HTRA1	HTRA1	25.038	22.560	0.000515	-2.478
Q9Y4L1	Hypoxia up-regulated protein 1	HYOU1	22.545	30.983	0.000000	8.438
P05362	Intercellular adhesion molecule 1	ICAM1	22.020	24.955	0.000368	2.935
P13598	Intercellular adhesion molecule 2	ICAM2	24.369	26.236	0.002242	1.867
P48735	Isocitrate dehydrogenase [NADP], mitochondrial	IDH2	23.565	27.297	0.000579	3.732

Q16666	Gamma-interferon-inducible protein 16	IFI16	21.575	25.623	0.000552	4.049
P35858	Insulin-like growth factor-binding protein complex acid labile subunit	IGFALS	25.413	22.019	0.000482	-3.394
P01591	Immunoglobulin J chain	IGJ	29.278	26.536	0.000528	-2.742
Q12905	Interleukin enhancer-binding factor 2	ILF2	24.403	28.070	0.000608	3.667
Q12906	Interleukin enhancer-binding factor 3	ILF3	22.205	26.983	0.000000	4.777
Q16891	MICOS complex subunit MIC60	IMMT	0.000	27.054	0.000000	27.054
P12268;P12269	Inosine-5-monophosphate dehydrogenase 2	IMPDH2	22.750	25.549	0.000628	2.799
O00410	Importin-5	IPO5	22.868	27.731	0.000759	4.863
Q9EPL8;O95373	Importin-7	IPO7	22.970	25.041	0.002048	2.071
P46940	Ras GTPase-activating-like protein IQGAP1	IQGAP1	27.579	28.968	0.006053	1.389
P17301	Integrin alpha-2	ITGA2	28.386	30.627	0.000750	2.241
P08648	Integrin alpha-5;Integrin alpha-5 heavy chain;Integrin alpha-5 light chain	ITGA5	27.422	29.601	0.001374	2.180
P23229	Integrin alpha-6;Integrin alpha-6 heavy chain;Integrin alpha-6 light chain;Processed integrin alpha-6	ITGA6	23.258	27.711	0.000484	4.453
P06756	Integrin alpha-V;Integrin alpha-V heavy chain;Integrin alpha-V light chain	ITGAV	27.340	29.921	0.000618	2.581
P05556	Integrin beta-1	ITGB1	29.092	31.445	0.000609	2.352
P05106	Integrin beta-3	ITGB3	27.488	28.798	0.009978	1.310
P14923	Junction plakoglobin	JUP	23.405	25.285	0.001914	1.880
Q15046	Lysine--tRNA ligase	KARS	23.705	25.906	0.001545	2.201
Q96CX2;Q6WVG3	BTB/POZ domain-containing protein KCTD12	KCTD12	23.656	26.135	0.000659	2.478
Q07666	KH domain-containing, RNA-binding, signal transduction-associated protein 1	KHDRBS1	22.934	26.442	0.000489	3.508
Q92945	Far upstream element-binding protein 2	KHSRP	21.329	24.605	0.000399	3.276
P33176	Kinesin-1 heavy chain	KIF5B	24.756	26.150	0.007777	1.394
O00505;O35344	Importin subunit alpha-4	KPNA3	0.000	22.739	0.003100	22.739
Q14974	Importin subunit beta-1	KPNB1	25.943	27.846	0.001904	1.903
P11279	Lysosome-associated membrane glycoprotein 1	LAMP1	26.466	29.302	0.000587	2.835
P13473	Lysosome-associated membrane glycoprotein 2	LAMP2	22.847	25.441	0.000588	2.594
Q14847	LIM and SH3 domain protein 1	LASP1	22.101	24.960	0.000391	2.859

P06151	L-lactate dehydrogenase A chain	LDHA	24.790	20.887	0.000725	-3.903
P00338;Q5R1W9	L-lactate dehydrogenase A chain	LDHA	26.690	29.404	0.000398	2.714
P07195;A5A6N7	L-lactate dehydrogenase B chain	LDHB	28.263	29.665	0.005170	1.402
Q32P28	Prolyl 3-hydroxylase 1	LEPRE1	17.389	27.007	0.000000	9.619
P09382	Galectin-1	LGALS1	28.069	31.359	0.000584	3.291
P49257	Protein ERGIC-53	LMAN1	18.514	28.842	0.000000	10.328
Q12907	Vesicular integral-membrane protein VIP36	LMAN2	24.091	28.804	0.000243	4.713
P02545;P48678	Prelamin-A/C;Lamin-A/C	LMNA	28.338	30.870	0.000642	2.532
P20700	Lamin-B1	LMNB1	21.170	26.424	0.000721	5.255
P36776	Lon protease homolog, mitochondrial	LONP1	20.761	26.801	0.000000	6.039
Q9Y4K0	Lysyl oxidase homolog 2	LOXL2	28.498	25.750	0.000404	-2.748
Q07954	Prolow-density lipoprotein receptor-related protein 1;Low-density lipoprotein receptor-related protein 1 85 kDa subunit;Low-density lipoprotein receptor-related protein 1 515 kDa subunit;Low-density lipoprotein receptor-related protein 1 intracellular domain	LRP1	28.077	24.876	0.000407	-3.201
Q96AG4	Leucine-rich repeat-containing protein 59	LRRC59	23.557	27.845	0.000217	4.288
O95777	U6 snRNA-associated Sm-like protein LSm8	LSM8	20.705	23.169	0.000641	2.463
P09960	Leukotriene A-4 hydrolase	LTA4H	22.279	25.764	0.000589	3.486
O75608	Acyl-protein thioesterase 1	LYPLA1	23.534	25.469	0.001815	1.935
O95372	Acyl-protein thioesterase 2	LYPLA2	23.585	26.218	0.000513	2.633
P20645	Cation-dependent mannose-6-phosphate receptor	M6PR	21.382	25.703	0.000729	4.321
Q16706	Alpha-mannosidase 2	MAN2A1	22.412	25.304	0.000370	2.892
P29678;Q02750;P36507	Dual specificity mitogen-activated protein kinase kinase 1;Dual specificity mitogen-activated protein kinase kinase 2	MAP2K1	28.620	26.189	0.000520	-2.431
P28482	Mitogen-activated protein kinase 1	MAPK1	23.520	25.975	0.000521	2.455

Q15691	Microtubule-associated protein RP/EB family member 1	MAPRE1	23.892	26.119	0.000584	2.227
Q15555	Microtubule-associated protein RP/EB family member 2	MAPRE2	24.999	22.601	0.000586	-2.398
P49006	MARCKS-related protein	MARCKSL1	23.550	25.560	0.001515	2.010
P48740	Mannan-binding lectin serine protease 1;Mannan-binding lectin serine protease 1 heavy chain;Mannan-binding lectin serine protease 1 light chain	MASP1	26.165	23.563	0.000389	-2.602
Q9NZL9	Methionine adenosyltransferase 2 subunit beta	MAT2B	21.824	23.841	0.003123	2.017
P43243	Matrin-3	MATR3	21.835	24.087	0.000850	2.252
P43121	Cell surface glycoprotein MUC18	MCAM	27.340	29.032	0.003656	1.692
P40925;P14152	Malate dehydrogenase, cytoplasmic	MDH1	24.816	27.385	0.000526	2.570
P40926	Malate dehydrogenase, mitochondrial	MDH2	25.310	30.159	0.000000	4.849
P23368	NAD-dependent malic enzyme, mitochondrial	ME2	19.659	26.474	0.000000	6.815
P55001	Microfibrillar-associated protein 2	MFAP2	19.066	28.147	0.000000	9.081
P08493;P08494	Matrix Gla protein	MGP	24.301	20.671	0.000555	-3.630
P14174	Macrophage migration inhibitory factor	MIF	23.196	26.961	0.000677	3.765
Q14165	Malectin	MLEC	20.845	27.189	0.000000	6.344
Q13201	Multimerin-1;Platelet glycoprotein Ia*;155 kDa platelet multimerin	MMRN1	27.640	25.848	0.002061	-1.792
Q96T76	MMS19 nucleotide excision repair protein homolog	MMS19	20.352	21.700	0.008631	1.347
O75352	Mannose-P-dolichol utilization defect 1 protein	MPDU1	0.000	26.218	0.000228	26.218
Q9UBG0	C-type mannose receptor 2	MRC2	25.600	22.396	0.000516	-3.204
Q92552	28S ribosomal protein S27, mitochondrial	MRPS27	27.852	24.413	0.000447	-3.439
P26038	Moesin	MSN	29.853	31.503	0.003474	1.650
P26927;Q2TV78	Hepatocyte growth factor-like protein;Hepatocyte growth factor-like protein alpha chain;Hepatocyte growth factor-like protein beta chain;Putative macrophage stimulating 1-like protein	MST1	25.883	24.020	0.003670	-1.863
Q13126	S-methyl-5-thioadenosine phosphorylase	MTAP	22.481	24.770	0.000749	2.289
P00403;P50690	Cytochrome c oxidase subunit 2	MT-CO2	23.004	28.301	0.000717	5.297
P58546	Myotrophin	MTPN	24.076	26.630	0.000529	2.554

P35580	Myosin-10	MYH10	24.302	26.787	0.001821	2.484
P35579	Myosin-9	MYH9	31.334	32.760	0.005282	1.425
P19105;O14950	Myosin regulatory light chain 12A;Myosin regulatory light chain 12B	MYL12A	28.084	30.293	0.000632	2.209
P60660	Myosin light polypeptide 6	MYL6	28.219	30.434	0.000658	2.215
O00159	Unconventional myosin-Ic	MYO1C	23.510	26.764	0.000635	3.254
Q9NZM1	Myoferlin	MYOF	22.622	28.898	0.000235	6.276
P43490	Nicotinamide phosphoribosyltransferase	NAMPT	24.633	28.443	0.000614	3.810
Q99733	Nucleosome assembly protein 1-like 4	NAP1L4	23.576	25.104	0.004374	1.527
P54920	Alpha-soluble NSF attachment protein	NAPA	21.071	25.978	0.000000	4.907
O43776	Asparagine--tRNA ligase, cytoplasmic	NARS	24.293	26.044	0.002104	1.751
P13591	Neural cell adhesion molecule 1	NCAM1	24.593	22.225	0.000607	-2.367
Q92542	Nicastrin	NCSTN	0.000	24.915	0.000636	24.915
P51970	NADH dehydrogenase [ubiquinone] 1 alpha subcomplex subunit 8	NDUFA8	0.000	26.220	0.000000	26.220
Q16795	NADH dehydrogenase [ubiquinone] 1 alpha subcomplex subunit 9, mitochondrial	NDUFA9	18.508	26.825	0.000000	8.317
P28331	NADH-ubiquinone oxidoreductase 75 kDa subunit, mitochondrial	NDUFS1	22.318	27.978	0.000000	5.660
O75306	NADH dehydrogenase [ubiquinone] iron-sulfur protein 2, mitochondrial	NDUFS2	0.000	25.874	0.000437	25.874
P48681	Nestin	NES	0.000	26.036	0.000470	26.036
P14543	Nidogen-1	NID1	24.308	21.999	0.000634	-2.310
P15531	Nucleoside diphosphate kinase A	NME1	26.074	29.037	0.000451	2.962
P22392;O60361	Nucleoside diphosphate kinase B;Putative nucleoside diphosphate kinase	NME2	23.403	27.649	0.000597	4.247
P40261	Nicotinamide N-methyltransferase	NNMT	21.881	24.823	0.000573	2.942
P69849;Q5JPE7	Nodal modulator 3;Nodal modulator 2	NOMO3	22.491	28.324	0.000000	5.832
Q15233	Non-POU domain-containing octamer-binding protein	NONO	23.139	26.346	0.000385	3.207
P61916	Epididymal secretory protein E1	NPC2	20.163	27.266	0.000000	7.103
P55786	Puromycin-sensitive aminopeptidase	NPEPPS	24.001	25.987	0.003104	1.986
Q9Y639	Neuroplastin	NPTN	22.031	25.386	0.000587	3.355
P15559	NAD(P)H dehydrogenase [quinone] 1	NQO1	22.692	24.279	0.006003	1.588
O43809	Cleavage and polyadenylation specificity factor subunit 5	NUDT21	0.000	23.486	0.003453	23.486

Q9NZJ9	Diphosphoinositol polyphosphate phosphohydrolase 2	NUDT4	20.553	23.571	0.000509	3.018
Q9UKK9	ADP-sugar pyrophosphatase	NUDT5	22.141	25.088	0.000354	2.947
Q8N1F7	Nuclear pore complex protein Nup93	NUP93	25.446	22.968	0.000664	-2.478
P61970	Nuclear transport factor 2	NUTF2	25.996	27.410	0.004821	1.414
Q56VL3	OCIA domain-containing protein 2	OCIAD 2	18.874	26.975	0.000000	8.102
Q02218	2-oxoglutarate dehydrogenase, mitochondrial	OGDH	0.000	26.105	0.000518	26.105
P20774	Mimecan	OGN	25.585	22.141	0.000358	-3.444
Q9NTK5	Obg-like ATPase 1	OLA1	23.596	25.515	0.001617	1.919
Q96FW1	Ubiquitin thioesterase OTUB1	OTUB1	23.198	26.047	0.000674	2.849
P13674	Prolyl 4-hydroxylase subunit alpha-1	P4HA1	18.466	27.661	0.000000	9.195
O15460	Prolyl 4-hydroxylase subunit alpha-2	P4HA2	18.428	27.438	0.000000	9.010
P07237	Protein disulfide-isomerase	P4HB	27.854	33.326	0.000000	5.472
Q9UQ80	Proliferation-associated protein 2G4	PA2G4	24.907	27.689	0.000392	2.782
P11940	Polyadenylate-binding protein 1	PABPC 1	24.998	26.854	0.001918	1.856
Q9UNF0	Protein kinase C and casein kinase substrate in neurons protein 2	PACSI N2	21.910	24.083	0.001540	2.173
P63005;P43034	Platelet-activating factor acetylhydrolase IB subunit alpha	PAFAH 1B1	22.660	24.499	0.002615	1.840
P68402	Platelet-activating factor acetylhydrolase IB subunit beta	PAFAH 1B2	24.079	26.068	0.001926	1.990
P22234	Multifunctional protein ADE2;Phosphoribosylaminoi midazole-succinocarboxamide synthase;Phosphoribosylamino imidazole carboxylase	PAICS	24.020	26.267	0.002013	2.248
Q99497	Protein DJ-1	PARK7	24.396	27.383	0.000466	2.987
Q9UKK3	Poly [ADP-ribose] polymerase 4	PARP4	22.601	21.112	0.005152	-1.489
Q9NVD7	Alpha-parvin	PARV A	20.131	23.772	0.000688	3.641
Q9HBI1	Beta-parvin	PARV B	25.366	23.625	0.003640	-1.741
Q15365	Poly(rC)-binding protein 1	PCBP1	25.549	27.342	0.003452	1.793
Q15366;P57721	Poly(rC)-binding protein 2;Poly(rC)-binding protein 3	PCBP2	23.811	26.389	0.000401	2.578
Q08174	Protocadherin-1	PCDH1	20.932	23.599	0.000387	2.666
Q9NPG4;O1491 7	Protocadherin-12;Protocadherin-17	PCDH1 2	22.685	0.000	0.008584	-22.685

P22061;P15246	Protein-L-isoaspartate(D-aspartate) O-methyltransferase	PCMT1	21.508	26.134	0.000218	4.625
Q9DEA3;P12004	Proliferating cell nuclear antigen	PCNA	22.477	26.161	0.000647	3.684
Q9UHG3	Prenylcysteine oxidase 1	PCYOX1	20.277	27.226	0.000000	6.950
Q8NBM8	Prenylcysteine oxidase-like	PCYOX1L	0.000	23.942	0.001974	23.942
P30101	Protein disulfide-isomerase A3	PDIA3	27.795	33.169	0.000000	5.373
P13667	Protein disulfide-isomerase A4	PDIA4	21.921	32.007	0.000000	10.086
Q14554	Protein disulfide-isomerase A5	PDIA5	21.068	27.449	0.000000	6.381
Q15084	Protein disulfide-isomerase A6	PDIA6	25.411	31.874	0.000000	6.463
O00151	PDZ and LIM domain protein 1	PDLIM1	23.306	24.944	0.005829	1.638
Q96HC4	PDZ and LIM domain protein 5	PDLIM5	21.331	26.089	0.000767	4.758
Q9NR12	PDZ and LIM domain protein 7	PDLIM7	0.000	24.205	0.000665	24.205
P30086	Phosphatidylethanolamine-binding protein 1;Hippocampal cholinergic neurostimulating peptide	PEBP1	24.102	27.652	0.000427	3.550
P16284	Platelet endothelial cell adhesion molecule	PECAM1	27.903	30.595	0.000346	2.692
O97239	Protein dopey homolog PFC0245c	PFC0245c	25.159	23.537	0.004380	-1.622
Q99471	Prefoldin subunit 5	PFDN5	0.000	24.607	0.000512	24.607
P07737	Profilin-1	PFN1	27.835	30.480	0.000633	2.645
P35080	Profilin-2	PFN2	22.115	23.540	0.006018	1.425
P18669	Phosphoglycerate mutase 1	PGAM1	27.219	29.022	0.002296	1.803
P52209	6-phosphogluconate dehydrogenase, decarboxylating	PGD	26.300	28.384	0.000852	2.083
A5A6K4;P00558	Phosphoglycerate kinase 1	PGK1	28.238	30.075	0.001788	1.837
O95336	6-phosphogluconolactonase	PGLS	0.000	25.859	0.000702	25.859
P36871	Phosphoglucomutase-1	PGM1	23.860	22.025	0.002061	-1.835
O00264	Membrane-associated progesterone receptor component 1	PGRMC1	0.000	26.445	0.000480	26.445
O15173	Membrane-associated progesterone receptor component 2	PGRMC2	0.000	26.501	0.000502	26.501
P35232	Prohibitin	PHB	20.776	30.166	0.000000	9.391
Q99623;O35129	Prohibitin-2	PHB2	18.324	29.725	0.000000	11.401
O00625	Pirin	PIR	21.188	24.080	0.000396	2.892
Q5JRX3	Presequence protease, mitochondrial	PITRM1	0.000	28.482	0.000000	28.482
P52480	Pyruvate kinase PKM	PKM	26.176	22.673	0.000644	-3.503
P00750	Tissue-type plasminogen activator;Tissue-type plasminogen activator chain A;Tissue-type plasminogen activator chain B	PLAT	22.662	20.587	0.001961	-2.075

Q03405	Urokinase plasminogen activator surface receptor	PLAUR	23.332	24.706	0.008493	1.374
Q8NHP8	Putative phospholipase B-like 2;Putative phospholipase B-like 2 32 kDa form;Putative phospholipase B-like 2 45 kDa form	PLBD2	19.005	25.699	0.000000	6.693
Q15149;P30427	Plectin	PLEC	23.939	29.152	0.000000	5.214
P08567	Pleckstrin	PLEK	25.885	22.928	0.000473	-2.957
Q02809	Procollagen-lysine,2-oxoglutarate 5-dioxygenase 1	PLOD1	25.541	29.863	0.000226	4.322
O00469	Procollagen-lysine,2-oxoglutarate 5-dioxygenase 2	PLOD2	21.572	29.145	0.000000	7.574
P13797	Plastin-3	PLS3	27.075	30.004	0.000389	2.929
Q6UX71	Plexin domain-containing protein 2	PLXDC2	23.534	19.672	0.000419	-3.862
Q9Y4D7	Plexin-D1	PLXND1	23.149	26.370	0.000384	3.221
O15305	Phosphomannomutase 2	PMM2	21.203	22.673	0.004797	1.470
P00491	Purine nucleoside phosphorylase	PNP	25.625	28.861	0.000567	3.236
O00592	Podocalyxin	PODXL	23.489	26.111	0.001537	2.622
P27170;P27169;Q91090;Q90952	Serum paraoxonase/arylesterase 1	PON1	25.129	22.796	0.000742	-2.333
Q15165	Serum paraoxonase/arylesterase 2	PON2	0.000	28.683	0.000000	28.683
Q15181	Inorganic pyrophosphatase	PPA1	20.869	27.319	0.000000	6.450
P62937	Peptidyl-prolyl cis-trans isomerase A;Peptidyl-prolyl cis-trans isomerase A, N-terminally processed	PPIA	28.834	31.178	0.000617	2.344
P23284	Peptidyl-prolyl cis-trans isomerase B	PPIB	25.821	31.228	0.000000	5.407
P49593	Protein phosphatase 1F	PPM1F	22.422	26.207	0.000475	3.786
P30153	Serine/threonine-protein phosphatase 2A 65 kDa regulatory subunit A alpha isoform	PPP2R1A	26.599	28.200	0.003456	1.601
P50897	Palmitoyl-protein thioesterase 1	PPT1	19.276	26.082	0.000000	6.806
O60831	PRA1 family protein 2	PRAF2	20.020	24.724	0.000000	4.703
P42785	Lysosomal Pro-X carboxypeptidase	PRCP	25.736	29.483	0.000617	3.747
Q06830	Peroxiredoxin-1	PRDX1	28.407	30.709	0.000649	2.302
P32119	Peroxiredoxin-2	PRDX2	25.626	27.778	0.000613	2.153
P30048	Thioredoxin-dependent peroxide reductase, mitochondrial	PRDX3	18.752	28.860	0.000000	10.108
Q13162;O08807	Peroxiredoxin-4	PRDX4	19.924	29.789	0.000000	9.866
P30044	Peroxiredoxin-5, mitochondrial	PRDX5	19.625	27.080	0.000000	7.455
P30041	Peroxiredoxin-6	PRDX6	26.064	27.863	0.002023	1.800
Q9JM99	Proteoglycan 4;Proteoglycan 4 C-terminal part	PRG4	25.332	23.290	0.002016	-2.042
P10644	cAMP-dependent protein kinase type I-alpha regulatory	PRKAR1A	23.076	25.197	0.001036	2.120

	subunit;cAMP-dependent protein kinase type I-alpha regulatory subunit, N-terminally processed					
P13861	cAMP-dependent protein kinase type II-alpha regulatory subunit	PRKAR2A	22.393	25.146	0.000345	2.753
P14314	Glucosidase 2 subunit beta	PRKCSH	20.380	30.226	0.000000	9.846
Q9UNN8	Endothelial protein C receptor	PROCR	21.667	23.562	0.003008	1.894
O95084	Serine protease 23	PRSS23	29.006	26.606	0.001016	-2.400
P07602	Prosaposin;Saposin-A;Saposin-B-Val;Saposin-B;Saposin-C;Saposin-D	PSAP	22.259	28.762	0.000000	6.503
O75832;Q9Z2X2	26S proteasome non-ATPase regulatory subunit 10	PSMD10	22.589	24.526	0.001901	1.938
P55036	26S proteasome non-ATPase regulatory subunit 4	PSMD4	23.433	25.394	0.002698	1.960
Q06323	Proteasome activator complex subunit 1	PSME1	22.784	25.861	0.000376	3.077
Q9UL46	Proteasome activator complex subunit 2	PSME2	22.846	25.887	0.000401	3.041
P26599	Polypyrimidine tract-binding protein 1	PTBP1	22.980	27.720	0.000229	4.739
Q9N0A4;Q9H7Z7	Prostaglandin E synthase 2;Prostaglandin E synthase 2 truncated form	PTGES2	0.000	24.390	0.000646	24.390
P20962	Parathymosin	PTMS	21.370	25.053	0.000606	3.682
P26022	Pentraxin-related protein PTX3	PTX3	28.974	25.916	0.000477	-3.058
P15151	Poliovirus receptor	PVR	22.284	23.970	0.004000	1.686
P49024;P49023	Paxillin	PXN	21.688	23.422	0.004105	1.733
P06737	Glycogen phosphorylase, liver form	PYGL	27.106	25.605	0.004392	-1.500
P47897	Glutamine--tRNA ligase	QARS	23.350	25.174	0.003651	1.825
O00391	Sulfhydryl oxidase 1	QSOX1	24.425	21.941	0.000519	-2.484
P62820	Ras-related protein Rab-1A	RAB1A	24.115	26.053	0.001809	1.937
P61019	Ras-related protein Rab-2A	RAB2A	23.779	25.627	0.002098	1.849
P51148	Ras-related protein Rab-5C	RAB5C	25.145	26.880	0.002759	1.735
P20340;Q9NRW1	Ras-related protein Rab-6A;Ras-related protein Rab-6B	RAB6A	24.366	25.799	0.005995	1.433
P54727	UV excision repair protein RAD23 homolog B	RAD23B	21.808	24.658	0.000362	2.850
P11233	Ras-related protein Ral-A	RALA	26.321	28.114	0.002019	1.793
P11234	Ras-related protein Ral-B	RALB	22.094	24.352	0.000651	2.258
P43487	Ran-specific GTPase-activating protein	RANBP1	0.000	25.607	0.000605	25.607
P46060	Ran GTPase-activating protein 1	RANGAP1	21.840	25.283	0.000465	3.443
P61225	Ras-related protein Rap-2b	RAP2B	22.878	24.287	0.006542	1.410
Q5U651	Ras-interacting protein 1	RASIP1	23.412	24.841	0.005103	1.429
Q09028	Histone-binding protein RBBP4	RBBP4	0.000	26.470	0.000452	26.470
A5A6M3;P38159	RNA-binding motif protein, X chromosome;RNA-binding	RBMX	21.681	25.809	0.000742	4.128

	motif protein, X chromosome, N-terminally processed					
P02753	Retinol-binding protein 4;Plasma retinol-binding protein(1-182);Plasma retinol-binding protein(1-181);Plasma retinol-binding protein(1-179);Plasma retinol-binding protein(1-176)	RBP4	26.961	25.229	0.003506	-1.732
P18754	Regulator of chromosome condensation	RCC1	22.970	24.885	0.001905	1.915
Q15293	Reticulocalbin-1	RCN1	20.213	30.942	0.000000	10.729
Q96D15	Reticulocalbin-3	RCN3	22.524	28.546	0.000000	6.022
P35241	Radixin	RDX	21.797	23.373	0.005075	1.576
P46063	ATP-dependent DNA helicase Q1	RECQL	23.258	25.370	0.001824	2.112
Q62159;P08134;Q6DHE8	Rho-related GTP-binding protein RhoC	RHOC	26.240	27.750	0.004386	1.509
P13489;Q8HZP9	Ribonuclease inhibitor	RNH1	22.743	28.100	0.000237	5.357
P30050	60S ribosomal protein L12	RPL12	26.559	28.302	0.002693	1.744
P35268;Q98TF8	60S ribosomal protein L22	RPL22	25.994	28.056	0.003003	2.062
P62829	60S ribosomal protein L23	RPL23	25.682	27.179	0.007256	1.497
P62750	60S ribosomal protein L23a	RPL23A	23.460	26.868	0.000522	3.408
P62899	60S ribosomal protein L31	RPL31	22.086	26.650	0.000216	4.564
Q98TF7;P42766	60S ribosomal protein L35	RPL35	23.427	27.177	0.000620	3.751
P61513	60S ribosomal protein L37a	RPL37A	23.019	25.495	0.000673	2.475
Q8HXB9;P63173	60S ribosomal protein L38	RPL38	24.693	26.888	0.000615	2.195
P05388;P14869;Q8NHW5	60S acidic ribosomal protein P0;60S acidic ribosomal protein P0-like	RPLP0	26.665	28.329	0.003448	1.664
P05386	60S acidic ribosomal protein P1	RPLP1	27.232	28.789	0.003876	1.557
P05387	60S acidic ribosomal protein P2	RPLP2	27.713	29.646	0.001910	1.934
P04843	Dolichyl-diphosphooligosaccharide--protein glycosyltransferase subunit 1	RPN1	20.852	30.594	0.000000	9.742
P04844	Dolichyl-diphosphooligosaccharide--protein glycosyltransferase subunit 2	RPN2	20.858	30.597	0.000000	9.739
P46783	40S ribosomal protein S10	RPS10	25.582	27.133	0.004465	1.551
P25398	40S ribosomal protein S12	RPS12	26.575	28.622	0.002054	2.047
P62844;P62841	40S ribosomal protein S15	RPS15	24.858	26.542	0.005254	1.684
P39019	40S ribosomal protein S19	RPS19	26.537	28.095	0.003715	1.558
P63220	40S ribosomal protein S21	RPS21	23.896	26.346	0.003667	2.450
P62857	40S ribosomal protein S28	RPS28	23.270	25.746	0.002689	2.476
P62081	40S ribosomal protein S7	RPS7	26.871	28.306	0.006718	1.435
O95197;Q9ES97	Reticulon-3	RTN3	24.016	26.841	0.000350	2.826

Q9NQC3;Q99P72	Reticulon-4	RTN4	25.901	28.521	0.000371	2.620
P31949	Protein S100-A11;Protein S100-A11, N-terminally processed	S100A11	27.794	29.766	0.001980	1.972
Q99584	Protein S100-A13	S100A13	22.396	25.375	0.000400	2.979
Q96FQ6	Protein S100-A16	S100A16	23.340	25.350	0.001911	2.011
P06703;P14069	Protein S100-A6	S100A6	27.352	29.268	0.004213	1.916
Q9UBE0;Q9R1T2	SUMO-activating enzyme subunit 1;SUMO-activating enzyme subunit 1, N-terminally processed	SAE1	20.746	24.741	0.000626	3.996
Q9Y512	Sorting and assembly machinery component 50 homolog	SAMM50	24.328	22.461	0.002870	-1.867
Q9NR31;Q9Y6B6	GTP-binding protein SAR1a;GTP-binding protein SAR1b	SAR1A;SAR1B	24.068	25.633	0.005082	1.565
Q14108	Lysosome membrane protein 2	SCARB2	24.990	28.691	0.000374	3.701
P22307	Non-specific lipid-transfer protein	SCP2	0.000	27.304	0.000691	27.304
Q9BRK5	45 kDa calcium-binding protein	SDF4	21.977	24.461	0.000740	2.485
P31040;P31039	Succinate dehydrogenase [ubiquinone] flavoprotein subunit, mitochondrial	SDHA	17.126	28.221	0.000000	11.095
P67812	Signal peptidase complex catalytic subunit SEC11A	SEC11A	18.636	26.105	0.000000	7.469
P55735	Protein SEC13 homolog	SEC13	23.927	25.552	0.004398	1.625
Q15436	Protein transport protein Sec23A	SEC23A	24.011	25.489	0.008114	1.479
P53992	Protein transport protein Sec24C	SEC24C	20.350	23.758	0.000526	3.408
O94855	Protein transport protein Sec24D	SEC24D	21.760	23.792	0.001429	2.033
O94979	Protein transport protein Sec31A	SEC31A	23.378	26.089	0.000570	2.710
Q9UGP8	Translocation protein SEC63 homolog	SEC63	0.000	25.022	0.000559	25.022
Q9NVA2	Septin-11	SEPT11	25.346	26.718	0.006274	1.372
Q15019	Septin-2	SEPT2	26.193	27.535	0.007886	1.342
Q8NC51	Plasminogen activator inhibitor 1 RNA-binding protein	SERBP1	21.809	25.030	0.000429	3.220
P35237	Serpin B6	SERPINB6	0.000	25.601	0.000592	25.601
P50453	Serpin B9	SERPINB9	22.170	26.934	0.000222	4.764
P05121	Plasminogen activator inhibitor 1	SERPINE1	30.151	28.760	0.006281	-1.391
P50454	Serpin H1	SERPINH1	27.317	32.166	0.000000	4.848
Q01105;P0DME0	Protein SET;Protein SETSIP	SET	25.948	27.651	0.005417	1.704
Q15637;Q64213	Splicing factor 1	SF1;Sf1	19.523	23.541	0.000595	4.018

Q12874	Splicing factor 3A subunit 3	SF3A3	22.574	24.443	0.003381	1.869
P31947	14-3-3 protein sigma	SFN	24.647	26.696	0.001611	2.049
P23246	Splicing factor, proline- and glutamine-rich	SFPQ	25.159	27.038	0.002755	1.879
Q9H9B4	Sideroflexin-1	SFXN1	0.000	27.767	0.000000	27.767
Q9H299;Q91VW3	SH3 domain-binding glutamic acid-rich-like protein 3	SH3BGRL3	21.980	25.415	0.000491	3.435
P34897	Serine hydroxymethyltransferase, mitochondrial	SHMT2	21.246	27.722	0.000000	6.476
P63208	S-phase kinase-associated protein 1	SKP1	21.590	24.483	0.000379	2.893
P53985	Monocarboxylate transporter 1	SLC16A1	22.951	25.027	0.001614	2.076
O15427	Monocarboxylate transporter 4	SLC16A3	22.517	24.401	0.003779	1.885
Q15758	Neutral amino acid transporter B(0)	SLC1A5	22.503	23.947	0.008501	1.443
Q00325;P12234	Phosphate carrier protein, mitochondrial	SLC25A3	20.422	30.037	0.000000	9.615
P51881;P05141	ADP/ATP translocase 2;ADP/ATP translocase 2, N-terminally processed	SLC25A5	20.003	29.565	0.000000	9.562
P12236	ADP/ATP translocase 3;ADP/ATP translocase 3, N-terminally processed	SLC25A6	25.878	31.514	0.000000	5.636
Q99808	Equilibrative nucleoside transporter 1	SLC29A1	22.967	24.623	0.005181	1.656
P08195	4F2 cell-surface antigen heavy chain	SLC3A2	25.609	27.567	0.002867	1.959
Q9GZT3	SRA stem-loop-interacting RNA-binding protein, mitochondrial	SLIRP	0.000	23.563	0.000413	23.563
O00161	Synaptosomal-associated protein 23	SNAP23	22.193	24.231	0.001968	2.038
Q7KZF4;Q66X93	Staphylococcal nuclease domain-containing protein 1	SND1	24.557	26.928	0.000743	2.370
P09012;P08579	U1 small nuclear ribonucleoprotein A;U2 small nuclear ribonucleoprotein B	SNRPA	22.804	25.042	0.000582	2.238
Q9UNH7	Sorting nexin-6;Sorting nexin-6, N-terminally processed	SNX6	21.751	23.175	0.005410	1.424
Q13813;P07751	Spectrin alpha chain, non-erythrocytic 1	SPTAN1	22.803	29.075	0.000250	6.271
Q01082	Spectrin beta chain, non-erythrocytic 1	SPTBN1	24.790	28.563	0.000548	3.773
P37108	Signal recognition particle 14 kDa protein	SRP14	22.788	25.373	0.000395	2.585
P49458	Signal recognition particle 9 kDa protein	SRP9	21.050	24.132	0.000441	3.081
Q07955;Q6NYA0;Q7SXP4	Serine/arginine-rich splicing factor 1;Serine/arginine-rich splicing factor 1B;Serine/arginine-rich splicing factor 1A	SRSF1	27.568	25.842	0.005183	-1.726
Q16629	Serine/arginine-rich splicing factor 7	SRSF7	25.557	23.765	0.003784	-1.792
P05455	Lupus La protein	SSB	23.796	26.748	0.000612	2.953

Q04837	Single-stranded DNA-binding protein, mitochondrial	SSBP1	24.834	26.956	0.000803	2.122
P43307	Translocon-associated protein subunit alpha	SSR1	19.708	27.761	0.000000	8.054
P51571	Translocon-associated protein subunit delta	SSR4	19.099	28.180	0.000000	9.081
P50502;Q8IZP2;Q8NFI4	Hsc70-interacting protein;Putative protein FAM10A4;Putative protein FAM10A5	ST13	25.206	26.461	0.009120	1.255
Q9NY15	Stabilin-1	STAB1	0.000	25.315	0.000221	25.315
Q13586	Stromal interaction molecule 1	STIM1	28.932	26.816	0.000583	-2.116
P31948	Stress-induced-phosphoprotein 1	STIP1	20.524	27.928	0.000000	7.404
Q9Y3F4	Serine-threonine kinase receptor-associated protein	STRAP	23.345	25.028	0.003457	1.684
P46978;P46977	Dolichyl-diphosphooligosaccharide--protein glycosyltransferase subunit STT3A	STT3A	21.594	28.228	0.000000	6.634
P53999	Activated RNA polymerase II transcriptional coactivator p15	SUB1	23.698	25.609	0.003686	1.911
O15260	Surfeit locus protein 4	SURF4	23.525	28.408	0.000000	4.883
Q9UH65	Switch-associated protein 70	SWAP70	21.886	24.658	0.000398	2.772
O60506;Q7TMK9	Heterogeneous nuclear ribonucleoprotein Q	SYNCRIP	24.244	27.351	0.000343	3.106
P37802	Transgelin-2	TAGLN2	28.657	30.432	0.002875	1.776
P37837	Transaldolase	TALDO1	24.960	26.872	0.001512	1.912
P26639;Q9D0R2	Threonine--tRNA ligase, cytoplasmic	TARS	20.716	26.410	0.000000	5.694
O75347	Tubulin-specific chaperone A	TBCA	22.038	25.520	0.000518	3.481
Q99426	Tubulin-folding cofactor B	TBCB	21.218	24.014	0.000388	2.796
Q15370	Transcription elongation factor B polypeptide 2	TCEB2	21.164	24.105	0.000392	2.941
P02786	Transferrin receptor protein 1;Transferrin receptor protein 1, serum form	TFRC	20.805	27.038	0.000000	6.234
Q15582	Transforming growth factor-beta-induced protein ig-h3	TGFBI	24.598	22.225	0.000594	-2.373
O43493	Trans-Golgi network integral membrane protein 2	TGOLN2	0.000	24.435	0.000352	24.435
P07996	Thrombospondin-1	THBS1	34.602	32.403	0.000602	-2.199
P35443	Thrombospondin-4	THBS4	28.010	25.204	0.000377	-2.807
P52888	Thimet oligopeptidase	THOP1	21.786	23.780	0.003847	1.994
Q9NQ88	Fructose-2,6-bisphosphatase TIGAR	TIGAR	22.125	24.426	0.000574	2.300
P29401	Transketolase	TKT	27.152	29.690	0.000363	2.538
Q99805	Transmembrane 9 superfamily member 2	TM9SF2	20.912	27.436	0.000000	6.524
Q92544	Transmembrane 9 superfamily member 4	TM9SF4	20.595	25.773	0.000000	5.178
P49755;O35587;Q63584;Q90515	Transmembrane emp24 domain-containing protein 10	TMED10	19.322	29.160	0.000000	9.838
Q15363	Transmembrane emp24 domain-containing protein 2	TMED2	24.637	26.753	0.001908	2.115

Q9Y3B3	Transmembrane emp24 domain-containing protein 7	TMED7	20.214	27.891	0.000000	7.677
Q9BVC6	Transmembrane protein 109	TMEM109	24.655	26.868	0.001818	2.214
P57088	Transmembrane protein 33	TMEM33	19.662	25.503	0.000000	5.840
Q9BTV4	Transmembrane protein 43	TMEM43	19.676	28.097	0.000000	8.421
P62328	Thymosin beta-4;Hematopoietic system regulatory peptide	TMSB4X	22.346	24.228	0.001994	1.882
P24821	Tenascin	TNC	24.262	20.643	0.000550	-3.620
P23510	Tumor necrosis factor ligand superfamily member 4	TNFSF4	24.256	26.649	0.001398	2.393
Q92973	Transportin-1	TNPO1	22.912	26.538	0.000541	3.626
Q15388	Mitochondrial import receptor subunit TOM20 homolog	TOMM20	21.583	23.595	0.001964	2.012
Q9NS69	Mitochondrial import receptor subunit TOM22 homolog	TOMM22	0.000	26.505	0.000220	26.505
O94826	Mitochondrial import receptor subunit TOM70	TOMM70A	20.799	26.341	0.000000	5.542
P60175;P60174;Q2QD07;P17751	Triosephosphate isomerase	TPI1	27.911	30.175	0.000590	2.264
P06753	Tropomyosin alpha-3 chain	TPM3	25.149	29.307	0.000737	4.158
P67936	Tropomyosin alpha-4 chain	TPM4	26.803	30.571	0.000733	3.769
O14773	Tripeptidyl-peptidase 1	TPP1	26.195	29.162	0.000393	2.967
P12270	Nucleoprotein TPR	TPR	20.742	23.307	0.000671	2.566
A5A6K2;P13693;P43348	Translationally-controlled tumor protein	TPT1	24.518	26.736	0.000649	2.218
Q14258	E3 ubiquitin/ISG15 ligase TRIM25	TRIM25	22.772	24.345	0.004766	1.573
Q7Z4G4	tRNA (guanine(10)-N2)-methyltransferase homolog	TRMT11	27.332	23.221	0.000746	-4.111
P10155;O08848	60 kDa SS-A/Ro ribonucleoprotein	TROVE2	20.737	24.535	0.000520	3.797
Q15631	Translin	TSN	21.945	23.945	0.001920	2.001
P30536	Translocator protein	TSPO	18.886	27.761	0.000000	8.874
P68366	Tubulin alpha-4A chain	TUBA4A	27.220	24.759	0.000670	-2.461
P49411	Elongation factor Tu, mitochondrial	TUFM	22.769	28.074	0.000000	5.305
Q6IBS0	Twinfilin-2	TWF2	23.933	25.991	0.001917	2.058
P10599	Thioredoxin	TXN	23.658	27.269	0.000569	3.611
O95881	Thioredoxin domain-containing protein 12	TXNDC12	22.977	26.456	0.000348	3.478
Q9BRA2	Thioredoxin domain-containing protein 17	TXNDC17	21.096	25.693	0.000231	4.597
Q8NBS9	Thioredoxin domain-containing protein 5	TXNDC5	27.311	32.910	0.000000	5.599
O43396	Thioredoxin-like protein 1	TXNL1	22.501	25.138	0.000630	2.637
Q16881	Thioredoxin reductase 1, cytoplasmic	TXNRD1	22.177	27.951	0.000000	5.773
P26369;P26368	Splicing factor U2AF 65 kDa subunit	U2AF2	23.790	26.210	0.000651	2.419
Q16222	UDP-N-acetylhexosamine pyrophosphorylase;UDP-N-	UAP1	0.000	22.042	0.007234	22.042

	acetylgalactosamine pyrophosphorylase;UDP-N-acetylglucosamine pyrophosphorylase					
P22314;Q02053	Ubiquitin-like modifier-activating enzyme 1	UBA1	27.684	29.199	0.003704	1.515
P62838;P62837;P61077;Q9Y2X8;P51668	Ubiquitin-conjugating enzyme E2 D2;Ubiquitin-conjugating enzyme E2 D3;Ubiquitin-conjugating enzyme E2 D4;Ubiquitin-conjugating enzyme E2 D1	UBE2D2	21.800	23.965	0.001403	2.165
P63280;P63279	SUMO-conjugating enzyme UBC9	UBE2I	22.315	25.551	0.000632	3.235
P68037;P68036	Ubiquitin-conjugating enzyme E2 L3	UBE2L3	23.797	26.875	0.000496	3.079
P61088;Q9FZ48;Q94A97	Ubiquitin-conjugating enzyme E2 N;Ubiquitin-conjugating enzyme E2 36;Ubiquitin-conjugating enzyme E2 35	UBE2N	22.381	26.374	0.000695	3.992
Q13404;Q15819	Ubiquitin-conjugating enzyme E2 variant 1;Ubiquitin-conjugating enzyme E2 variant 2	UBE2V1	22.229	25.642	0.000394	3.413
P09936	Ubiquitin carboxyl-terminal hydrolase isozyme L1	UCHL1	26.122	29.199	0.000500	3.077
Q9NYU2	UDP-glucose:glycoprotein glucosyltransferase 1	UGGT1	18.317	29.845	0.000000	11.528
Q91ZJ5;Q16851	UTP--glucose-1-phosphate uridylyltransferase	UGP2	23.791	25.595	0.003709	1.804
P31930	Cytochrome b-c1 complex subunit 1, mitochondrial	UQCRC1	21.221	29.460	0.000000	8.239
P22695	Cytochrome b-c1 complex subunit 2, mitochondrial	UQCRC2	20.433	29.294	0.000000	8.860
P06132	Uroporphyrinogen decarboxylase	UROD	22.181	24.373	0.000739	2.192
P54578;P60051	Ubiquitin carboxyl-terminal hydrolase 14	USP14	20.992	25.819	0.000000	4.827
P45974	Ubiquitin carboxyl-terminal hydrolase 5	USP5	22.421	25.133	0.000510	2.711
Q15836;P63027	Vesicle-associated membrane protein 3;Vesicle-associated membrane protein 2	VAMP3	22.195	23.776	0.004132	1.581
Q9P0L0	Vesicle-associated membrane protein-associated protein A	VAPA	19.007	26.249	0.000000	7.242
Q99536	Synaptic vesicle membrane protein VAT-1 homolog	VAT1	25.735	28.087	0.000580	2.352
P61758	Prefoldin subunit 3	VBP1	0.000	25.144	0.000585	25.144
P21796	Voltage-dependent anion-selective channel protein 1	VDAC1	26.060	29.522	0.000487	3.461
P45880	Voltage-dependent anion-selective channel protein 2	VDAC2	22.890	28.605	0.000000	5.714
P08670	Vimentin	VIM	30.929	34.411	0.000641	3.483
O75436	Vacuolar protein sorting-associated protein 26A	VPS26A	22.144	24.743	0.000638	2.599
Q9UBQ0;Q7ZV68	Vacuolar protein sorting-associated protein 29	VPS29	0.000	23.717	0.005150	23.717
P23381	Tryptophan--tRNA ligase, cytoplasmic;T1-TrpRS;T2-TrpRS	WARS	24.470	27.371	0.000386	2.902

O75083	WD repeat-containing protein 1	WDR1	28.167	29.522	0.008594	1.355
Q9NQW7	Xaa-Pro aminopeptidase 1	XPNPEP1	22.731	25.060	0.000631	2.329
O14980;Q80U96	Exportin-1	XPO1	24.794	26.145	0.007539	1.351
P67809	Nuclease-sensitive element-binding protein 1	YBX1	24.656	26.421	0.003371	1.766
P31946	14-3-3 protein beta/alpha;14-3-3 protein beta/alpha, N-terminally processed	YWHAB	26.449	28.319	0.001886	1.870
P62258	14-3-3 protein epsilon	YWHAE	27.860	29.871	0.001278	2.011
P61981	14-3-3 protein gamma;14-3-3 protein gamma, N-terminally processed	YWHAG	26.386	28.440	0.000751	2.054
Q04917	14-3-3 protein eta	YWHAH	23.634	26.075	0.000627	2.441
P27348	14-3-3 protein theta	YWHAQ	26.172	28.373	0.000623	2.201
P63104	14-3-3 protein zeta/delta	YWHAZ	27.945	29.782	0.001898	1.838
Q15942	Zyxin	ZYX	22.303	25.638	0.000539	3.334

Table S1. Proteins commonly expressed by eEVs and e-EV-IL-3. Data are expressed as LFQ intensity values that were log2 transformed, and proteins were considered significant if passed the *two sample t-test*, with the following settings; S0 = 2 and FDR 0.01. Delta positive values represent proteins up-regulated in eEV-IL-3 compared eEV and *vice versa* for negative values.

Table S2.

IL-8 signalling					
**Protein name**	**Gene names**	**LFQ intensity eEV (log2)**	**LFQ intensity eEV-IL-3 (log2)**	**Student's t-test q-value**	**Delta**
Angiopoietin-2	ANGPT2	24.10771	26.09117	0.001923	1.98346
Cystatin-B	CSTB	0	26.38522	0.000684	26.38522
Guanine nucleotide-binding protein G(i) subunit alpha-2	GNAI2	26.41312	28.61017	0.001395	2.19705
Guanine nucleotide-binding protein G(I)/G(S)/G(T) subunit beta-1	GNB1	26.49009	27.9145	0.005116	1.42441
Guanine nucleotide-binding protein G(I)/G(S)/G(O) subunit gamma-12	GNG12	23.70675	26.41369	0.000579	2.70694
Intercellular adhesion molecule 1	ICAM1	22.02013	24.9551	0.000368	2.93497
Ras GTPase-activating-like protein IQGAP1	IQGAP1	27.57858	28.96766	0.006053	1.38908
Integrin alpha-V;Integrin alpha-V heavy chain;Integrin alpha-V light chain	ITGAV	27.33958	29.92056	0.000618	2.58098
Integrin beta-3	ITGB3	27.48788	28.798	0.009978	1.31012
LIM and SH3 domain protein 1	LASP1	22.10136	24.95987	0.000391	2.85851
Dual specificity mitogen-activated protein kinase kinase 1;Dual specificity mitogen-activated protein kinase kinase 2	MAP2K1	28.62046	26.18923	0.00052	-2.43123
Mitogen-activated protein kinase 1	MAPK1	23.52009	25.97544	0.000521	2.45535
Ras-related protein Ral-A	RALA	26.32124	28.11421	0.002019	1.79297
Ras-related protein Ral-B	RALB	22.09353	24.35151	0.000651	2.25798
Ras-related protein Rap-2b	RAP2B	22.87766	24.2872	0.006542	1.40954
Rho-related GTP-binding protein RhoC	RHOC	26.24047	27.74979	0.004386	1.50932

Table S2. Mass spectrometry data of IL-8 pathway comparing eEV and eEV-IL-3. Data are expressed as LFQ intensity values that were log2 transformed, and proteins were considered significant if passed the *two sample t-test*, with the following settings; S0 = 2 and FDR 0.01. Delta positive values represent proteins up-regulated in eEV-IL-3 compared eEV and *vice versa* for negative values.

Table S3.

NF-kB Signaling					
**Protein name**	**Gene names**	**LFQ intensity eEV (log2)**	**LFQ intensity eEV-IL-3 (log2)**	**Student's t-test q-value**	**Delta**
Ras-related protein Ral-A	RALA	26.32124	28.11421	0.002019	1.79297
Ras-related protein Ral-B	RALB	22.09353	24.35151	0.000651	2.25798
Ras-related protein Rap-2b	RAP2B	22.87766	24.2872	0.006542	1.40954
Ubiquitin-conjugating enzyme E2 N;Ubiquitin-conjugating enzyme E2 36;Ubiquitin-conjugating enzyme E2 35	UBE2N	22.38121	26.37365	0.000695	3.99244
Ubiquitin-conjugating enzyme E2 variant 1;Ubiquitin-conjugating enzyme E2 variant 2	UBE2V1	22.22916	25.64214	0.000394	3.41298

Table S3. Mass spectrometry data of NF-kB pathway comparing eEV and eEV-IL-3. Data are expressed as LFQ intensity values that were log2 transformed, and proteins were considered significant if passed the *two sample t-test*, with the following settings; S0 = 2 and FDR 0.01. Delta positive values represent proteins up-regulated in eEV-IL-3 compared eEV and *vice versa* for negative values.

Table S4.

Protein names	Gene names	LFQ intensity eEV (log2)	LFQ intensity eEV-IL-3 (log2)	Student's t-test q-value	Delta
Intercellular adhesion molecule 1	ICAM1	22.02013	24.9551	0.000368	2.93497
Acidic leucine-rich nuclear phosphoprotein 32 family member A	ANP32A	21.38723	26.84077	0.000248	5.45354
Acidic leucine-rich nuclear phosphoprotein 32 family member B	ANP32B	22.69875	24.32486	0.004121	1.62611
Complement component 1 Q subcomponent-binding protein, mitochondrial	C1QBP	21.57465	28.51319	0.000247	6.93854
Complement C1q tumor necrosis factor-related protein 3	C1QTNF3	24.69608	21.06215	0.000508	-3.63393
Collagen alpha-1(XII) chain	COL12A1	28.64294	26.61961	0.003086	-2.02333
Collagen alpha-2(I) chain	COL1A2	25.72285	24.2686	0.00679	-1.45425
Collagen alpha-1(IV) chain;Arresten	COL4A1	24.32141	26.8397	0.002909	2.51829
Collagen alpha-1(VI) chain	COL6A1	28.06933	25.28857	0.000502	-2.78076
Collagen alpha-3(VI) chain	COL6A3	25.24042	22.75566	0.000637	-2.48476
Gamma-interferon-inducible protein 16	IFI16	21.57489	25.62347	0.000552	4.04858
Interleukin enhancer-binding factor 2	ILF2	24.40302	28.07029	0.000608	3.66727
Interleukin enhancer-binding factor 3	ILF3	22.20524	26.98262	0	4.77738
Lysyl oxidase homolog 2	LOXL2	28.49756	25.74974	0.000404	-2.74782
Tumor necrosis factor ligand superfamily member 4	TNFSF4	24.25648	26.64944	0.001398	2.39296

Table S4. **Mass spectrometry data of inflammatory related proteins identified in eEV and eEV-IL-3 compared with proteins identified in EV released by HUVEC in response to TNF-α (eEV-TNF-α)** [43]. Data are expressed as LFQ intensity values that were log2 transformed, and proteins were considered significant if passed the *two sample t-test*, with the following settings; S0 = 2 and FDR 0.01. Positive Delta positive values represent proteins up-regulated in eEV-IL-3 compared eEV and *vice versa* for negative values.
